# Redesigning Follow-Up Care in Community Mental Health Teams Through Patient-Initiated Models: A Quality Improvement Project

**DOI:** 10.1192/bjo.2026.11523

**Published:** 2026-06-30

**Authors:** Muhammad Zeeshan, Mohammed Yousef, Kallol Sain, Jovanpreet Singh Rai, Morouge Elbadawey

**Affiliations:** Birmingham and Solihull Mental Health NHS Foundation Trust, Birmingham, United Kingdom

## Abstract

**Aims:**

Traditional Community Mental Health Team (CMHT) follow-up relies on clinician scheduled appointments at fixed intervals, which may not be responsive to individual patient needs. Patient-Initiated Follow-Up (PIFU) enables patients to request timely review based on symptom change rather than routine scheduling. This project evaluated implementation of a PIFU pathway for a cohort of patients within Aston and Nechells CMHTs, with Aston acting as a control using the conventional pre-scheduled follow-up model and Nechells implementing PIFU, to assess the impact on access, capacity and patient experience.

**Methods:**

The pathway was designed using NHS England's published PIFU guidance and registered with the Trust’s Quality Improvement team. A dedicated PIFU category was created within the electronic patient record, clinicians were allocated weekly PIFU review slots, and relevant staff received training. Following clinical assessment, patients werescreened using agreed criteria to identify those with PIFU-suitable mental health presentations, adequate capacity and ability to self-initiate contact. The process was explained and informed consent obtained. All transfers to PIFU were discussed at multidisciplinary team meetings prior to implementation. Patients re-accessed care by contacting the CMHT directly and were offered appointments within seven days. Uptake of PIFU and its impact on routine appointment availability were monitored and compared between Nechells and Aston CMHTs. Vacant PIFU slots were repurposed for ad hoc reviews of patients attending Clozapine or depot clinic.

**Results:**

From February 2025 to May 2025, 183 patients had been placed on the PIFU pathway, with 31 PIFU reviews completed. In June 2025, Nechells CMHT demonstrated a 37.22% reduction in routine clinic waiting times compared with Aston CMHT, alongside improved capacity for review slots within 14 days. By December 2025, there were 311 patients on the PIFU pathway. Patient feedback demonstrated good understanding of the pathway, with 66.7% reporting clear understanding and 72.7% confidence in getting access. Since initiation, 31.5% of patients had contacted the team, with 68.2% finding access easy. Overall, 76.8% preferred PIFU and 92.6% wished to continue on the pathway, a small proportion expressed preference for the traditional model.

**Conclusion:**

A PIFU pathway within CMHT for selected patients resulted in significant reductions in waiting times, improved clinic capacity and gave favourable patient satisfaction. The model supports timely access to care while optimising resource use. Following the results of this project, formal standard operating procedure was developed and trust-wide rollout of PIFU across all CMHTs within Birmingham and Solihull Mental HealthFoundation Trust is planned with further evaluation.

